# Ten Simple Rules to Win a Nobel Prize

**DOI:** 10.1371/journal.pcbi.1004084

**Published:** 2015-04-02

**Authors:** Richard J. Roberts

**Affiliations:** New England Biolabs, Ipswich, Massachusetts, United States of America; National Institutes of Health, United States of America

**Preface**

by Philip E. Bourne, National Institutes of Health, Founding Editor-in-Chief of *PLOS Computational Biology*

When receiving a draft of the article “Ten Simple Rules for Writing a PLOS Ten Simple Rules Article” [1], not only had we come full circle in terms of professional development, but also I knew the series was a success. Since that article was published in October 2014, two more articles have been published, and this will be the third: a total of 44 in all. Rule 2 in what I shall affectionately call the 102 article [1] suggested you need a novel topic and suggested winning a Nobel Prize was such a topic. As I hinted in my editorial comments to the 102 article, I would take up the challenge in soliciting such an article. Rich Roberts was the first person to come to mind, partly because he is a good sport, partly because we share an interest in open (to be interpreted here as candid) science, and of course because he won the Nobel Prize in Physiology or Medicine (with Phillip Sharp) in 1993 for work on gene structure.

At first he was reluctant and slightly insulted, making me think I should write “Ten Simple Rules for How Not to Insult a Nobel Laureate.” The rationale is that we should not be encouraging scientists to think about science through awards but through having fun and the desire to do their best science. That should be enough. The result is exactly that—having a bit of fun and making some important points all at once. I hope you enjoy it as much as I did.

1. http://www.ploscollections.org/article/info:doi/10.1371/journal.pcbi.1003858

## Introduction

It is remarkable how many students, young faculty, and even senior faculty hanker after a Nobel Prize. Somehow, they think that it is possible to structure their scientific careers so that the culmination will bring this much sought-after honor. Some even think that as a Nobel laureate myself, I may have the key to success—some secrets that I can share and so greatly improve their odds of success. Unfortunately, I must begin by disappointing everyone. There is only one path that should be followed. It is summed up in Rule 1, but some of the other Rules may prove helpful—or if not helpful, then at least amusing.

### 1. Never Start Your Career by Aiming for a Nobel Prize

Don’t even hope for it or think about it. Just focus on doing the very best science that you can. Ask good questions, use innovative methods to answer them, and look for those unexpected results that may reveal some unexpected aspect of nature. If you are successful in your research career, then you will make lots of discoveries and have a very happy life. If you are lucky, you will make a big discovery that may even bag you a prize or two. But only if you are extraordinarily lucky will you stand any chance of winning a Nobel Prize. They are very elusive.

### 2. Hope That Your Experiments Fail Occasionally

There are usually two main reasons why experiments fail. Very often, it is because you screwed up in the design by not thinking hard enough about it ahead of time. Perhaps more often, it is because you were not careful enough in mixing the reagents (I always ask students if they spat in the tube or, more recently, were texting when they were labeling their tubes). Sometimes, you are not careful enough in performing the analytics (did you put the thermometer in upside down, as I once witnessed from a medical student whose name now appears on my list of doctors who I won’t allow to treat me even if I’m dying?). These problems are the easiest to deal with by always taking great care in designing and executing experiments. If they still fail, then do them over again! But the more interesting reason that experiments fail is because nature is trying to tell you that the axioms on which you based the experiment are wrong. This means the dogma in the field is wrong (often the case with dogma). If you are lucky, as I was, then the dogma will be seriously wrong, and you can design more experiments to find out why. If you are really lucky, then you will stumble onto something big enough to be prizeworthy.

### 3. Collaborate with Other Scientists, but Never with More Than Two Other People

Collaboration embodies much of what is good about science and makes it fun. By bringing different sets of expertise to bear on a problem, it is often the key to making discoveries. However, if you think you are getting close to a big discovery, always keep in the back of your mind that there can only be three winners on the ticket for a Nobel Prize. Pick your collaborators carefully! But seriously, don’t do as some have done and try to make a competitor of someone who would otherwise be an extremely valuable collaborator.

### 4. To Increase Your Odds of Winning, Be Sure to Pick Your Family Carefully

Seven children of Nobel Prize winners have gone on to win the Prize themselves, and four married couples have jointly won the Prize. Marie Curie and her husband, Pierre, won in Physics in 1903, while their daughter Irene with her husband, Frederic Joliot, won the Chemistry Prize in 1935. Carl and Gerty Cori won the Medicine Prize in 1947, and Alva Myrdal and Alfonso Robles won the Peace Prize in 1942. Lawrence Bragg shared the Physics Prize in 1935 with his father, William. Roger Kornberg (Chemistry, 2006) and his father, Arthur, (Medicine, 1959) both won. Aage Bohr (1975) and his father, Niels, (1922) both won the Physics Prize. Other father-son laureates are the Swedes Hans von Euler-Chelpin (Chemistry, 1929) and Ulf von Euler (Medicine, 1970) and Manne Siegbahn (1924) and Kai Siegbahn (1981), both in Physics. Briton Joseph John Thomson (1906) and his son George (1937) both won the Physics Prize. The only siblings to bask in Nobel glory were Jan and Nikolaas Tinbergen (Medicine, 1973) of the Netherlands. Jan won the first Prize awarded in Economics in 1969.

With a total of 586 Nobel Prize recipients in science during the 113 years since it was first awarded, these are impressive numbers, given a world population numbering at least 10,000,000,000 over the same period of time.

This rule is vividly illustrated last year (2014) by another married couple sharing the Nobel Prize in Physiology or Medicine.

### 5. Work in the Laboratory of a Previous Nobel Prize Winner

Many Prize recipients have benefitted greatly from the inspiration that this approach can bring. Sometimes just working at an institution with a previous Prize winner can be helpful. One prime example is the Medical Research Council (MRC) Laboratory in Cambridge, United Kingdom, where no less than nine staff members have won Nobel Prizes in either Chemistry or Physiology and Medicine, including my own personal hero Fred Sanger, who won the Chemistry Prize twice (1958, 1980), once for inventing protein sequencing and once for pioneering DNA sequencing. In between, he also invented RNA sequencing, but perhaps three Prizes was more than the Nobel Committee could stomach.

### 6. Even Better Than Rule 5, Try to Work in the Laboratory of a Future Nobel Prize Winner

This can be very beneficial, especially if you can be a part of the Prize-winning discovery. That has proven to be a very good strategy, but it is not always easy to spot the right mentor, one who will bring you that sort of success and then share the glory with you. The corollary of this strategy is not to work in the laboratory of someone who has already won but whom you think will win again with you on the ticket. This has yet to prove successful based on the previous double recipients named in Rule 5! It is much better to make sure that any big discoveries come from you after you leave the lab and are out on your own.

### 7. Always Design and Execute Your Best Experiments at a Time When Your Luck Is Running High

A casual survey of Nobel Prize winners will soon confirm that most credit luck as being the biggest component in their discovery. This is partly because many discoveries arise when what we think we know turns out to be wrong and we base our further research on incorrect assumptions. However, only rarely are we lucky enough to have to make such dramatic changes in our assumptions that a really major breakthrough becomes possible—the sort that may one day be considered appropriate for a Nobel Prize.

### 8. Never Plan Your Life around Winning a Nobel Prize

This has proven disastrous for many people. I know several scientists who became convinced that they were going to win and had all sorts of plans for less-than-modest speeches acknowledging the award of the Prize, preparing comments for journalists and planning subsequent trips to exotic places to talk about their discovery. It is far better not to know you have been nominated so that it comes as a real surprise when you get the early morning call from Stockholm. In fact, why not just forget about the Nobel Prize altogether and focus on doing the very best science you can? If you decide to ignore this rule, under no circumstances should you bug current Nobel laureates to nominate you. This has been an all-too-common strategy employed by many who feel they should be laureates, some even going so far as to send their last year’s publications along every year with a reminder of what they consider their “big” discovery. This will almost guarantee that the laureate won’t nominate you and is likely to lead to them advising their friends similarly. Can you imagine how that conversation would go after a few late-night drinks in the bar?

### 9. Always Be Nice to Swedish Scientists

Several laureates had their prize severely delayed by picking a fight with the wrong person, someone who was either already a Nobel Committee member or became one subsequent to the fight. Some individuals may even have lost out altogether, although one would need to search the archives (only available 50 years after the award) to find them. This is usually an easy rule to follow as in my experience the Swedes are very nice people, good scientists, easy to collaborate with, and extremely amiable drinking partners.

It is never too early to get started on this. Then, should your name magically appear on the candidates’ list and you have to wait for it to reach the top, you may still be around to cash in. Peyton Rous had to wait from 1911 until 1966 for the Medicine Prize, just four years before his death.

### 10. Study Biology

There are many reasons for this. First, biology is fascinating, never boring, and directly affects our everyday lives, yet we still know relatively little about it. Thus, the odds of making a big discovery are greatly increased compared to other disciplines. Second, biology is all around us, is vastly complicated, and encompasses disciplines such as medicine, agriculture, conservation, and computer science, as well as many others, thus lending itself to the kind of interdisciplinary approaches that make science such fun and can easily lead into new territory. Third, unlike physics and chemistry, biology is ever changing, thanks to evolution. What seems to be the rule today may have changed by the time you are doing your experiments. Finally, there are two Prize categories in which biological discoveries are currently being awarded. One is Physiology or Medicine, and the other is Chemistry, in which about half the Prizes go to biologists. Already you have increased your odds by 50%.

### Conclusions

In summary, Rule 1 is the best advice I can offer. There is no substitute for pursuing the very best science that you can. Even Marie Curie, John Bardeen, and Fred Sanger needed this to win their second Prize. In contrast, Linus Pauling, one of the cleverest chemists of his generation, only received his second Prize (in Peace) by working in a totally different field. Nevertheless, the odds of winning a second Prize, if you already have one, do seem rather better than average!

